# Genetic, transcriptional and post-translational regulation of the programmed death protein ligand 1 in cancer: biology and clinical correlations

**DOI:** 10.1038/s41388-018-0303-3

**Published:** 2018-05-16

**Authors:** Ioannis Zerdes, Alexios Matikas, Jonas Bergh, George Z. Rassidakis, Theodoros Foukakis

**Affiliations:** 1 Department of Oncology-Pathology, Cancer Centrum Karolinska, Karolinska Institutet, Stockholm, Sweden; 2 Department of Oncology, Radiumhemmet, Karolinska University Hospital, Stockholm, Sweden; 3 Department of Pathology and Cytology, Karolinska University Hospital, Stockholm, Sweden

## Abstract

The programmed death protein 1 (PD-1) and its ligand (PD-L1) represent a well-characterized immune checkpoint in cancer, effectively targeted by monoclonal antibodies that are approved for routine clinical use. The regulation of PD-L1 expression is complex, varies between different tumor types and occurs at the genetic, transcriptional and post-transcriptional levels. Copy number alterations of PD-L1 locus have been reported with varying frequency in several tumor types. At the transcriptional level, a number of transcriptional factors seem to regulate PD-L1 expression including HIF-1, STAT3, NF-κΒ, and AP-1. Activation of common oncogenic pathways such as JAK/STAT, RAS/ERK, or PI3K/AKT/MTOR, as well as treatment with cytotoxic agents have also been shown to affect tumoral PD-L1 expression. Correlative studies of clinical trials with PD-1/PD-L1 inhibitors have so far shown markedly discordant results regarding the value of PD-L1 expression as a marker of response to treatment. As the indications for immune checkpoint inhibition broaden, understanding the regulation of PD-L1 in cancer will be of utmost importance for defining its role as predictive marker but also for optimizing strategies for cancer immunotherapy. Here, we review the current knowledge of PD-L1 regulation, and its use as biomarker and as therapeutic target in cancer.

## Introduction

Cancer development and progression raises a strong antitumor immune response through which the immune system can eliminate cancer cells. This immunosurveillance theory describes the complex interactions between immune and cancer cells, divided in three distinct but often overlapping stages: elimination, equilibrium, and evasion. Thus, tumors can suppress immunity and escape eradication; evading immune destruction has been characterized as a hallmark of cancer [1, 2].

Programmed death protein 1 (PD-1) and its ligand (PD-L1) have been recognized as inhibitory molecules that cause impaired immune response against cancer cells. Therapeutic antibodies targeting PD-1/PD-L1 have been introduced into clinical practice, leading to better patient outcomes [3]. Immune checkpoint regulation has been under intense investigation over the last decades, however, the underlying mechanisms regulating the PD1 and PD-L1 expression are not fully understood; several oncogenic signaling pathways, epigenetic modifications, and genetic variations have been suggested. The aim of this review is to summarize the current knowledge on PD-L1 regulation and its emerging role as a target in cancer immunotherapy.

## Immune surveillance: the role of PD-1/PD-L1 axis as immune checkpoint

PD-1 (CD279) is a transmembrane protein, member of the CD28 family. It is mainly expressed on activated T cells but it can also be detected in other cells such as B- and natural killer (NK) cells upon induction [4]. PD-1 has two ligands, PD-L1 (CD274, B7-H1) and PD-L2 (CD273, B7-DC), which belong to the B7-CD28 protein family [5]. PD-L1 is expressed on tumor cells but it can also be present on the surface of other cell types including T cells, B cells, dendritic cells, macrophages, mesenchymal stem cells, epithelial, endothelial cells, and as recently shown, brown adipocytes [6]. PD-L2 is typically expressed in antigen-presenting cells (APCs). PD-L1 is expressed upon stimulation of cytokine interferon-γ (IFNg), secreted by activated T cells [7, 8].

PD-L1 and PD-L2 are encoded by the *CD274* and *PDCD1LG2* genes, respectively, located on chromosome 9p.24.1, whereas PD-1 is encoded by the *PDCD1* gene located on chromosome 2q37.3 [4].

PD-1/PD-L1 axis plays an important role in the regulation of T-cell immunity and has been also implicated in autoimmunity and infection [9]. The PD-1/PD-L1 interaction has been characterized as an “immune checkpoint” due to its impact on the orchestration of immune response against tumor antigens. Along with cytotoxic T-lymphocyte-associated protein 4 (CTLA-4, CD152), they represent immunological “brakes” that modulate T-cell activation leading to an impaired immunosurveillance.

T-cell activation involves a two signal-model; APCs require a first signal from T-cell receptor (TCR), which recognizes the antigen along with the major histocompatibility complex (MHC) presented on the surface of APC. The second signal includes the co-stimulatory interaction between CD28 on the surface of T cells and CD80 (B7.1) or CD86 (B7.2) on the surface of APC [10, 11].

The engagement of PD-1 with its ligands leads to the inhibition of T-cell activation and response, via mechanisms that include blocking of proliferation, induction of apoptosis, and regulatory T-cell differentiation and therefore immune inhibition [11]. Blocking the PD-1/PD-L1 axis with potent monoclonal antibodies may reverse the impaired anticancer immunity and thus represents an appealing target of cancer immunotherapy [12].

## The genetic basis of PD-L1 expression in cancer

The genetic aberrations of the PD-L1/PD-L2 gene loci represent a key mechanism of PD-L1 expression both in solid and hematologic tumors. Studies of copy number alterations (CNAs) have been reported in several tumor types (Table 1). The highest frequencies of CNAs have been seen in squamous cell carcinomas of vulva and cervix and triple-negative breast cancer (TNBC), as well as in classical Hodgkin lymphoma (cHL) and primary mediastinal B-cell lymphoma (PMBCL). Contrary, low or absent CNAs have been reported in small and non-small cell lung cancer (NSCLC) and in diffuse large B-cell lymphomas (DLBCL). In general, copy number gains and especially amplifications are well correlated with the protein levels of PD-L1. Given the challenges in determining the protein levels of PD-L1 as detailed below, detection of CNAs is an attractive alternative for identifying patients who could benefit from treatment with checkpoint inhibitors. Table 1 summarizes the current literature of the genetic regulation of PD-L1 [13–28]. In addition to these individual studies, a large in silico analysis of CNAs in PD-L1 has been conducted using the Cancer Genome Atlas datasets (22 cancer types, 9771 tumors). Interestingly, deletions of 9p24.1 were more common than gains in this analysis and were found mostly in melanoma and NSCLC, with gains occurring frequently in ovarian, head and neck, bladder, and cervical carcinomas [29].

**Table 1:** Copy number alterations (CNAs) of CD274 gene in cancer

Tumor type(s)	No. of cases	Method(s)	% of gains (*n*)	% Amplifications (*n*)	Association with IHC (PD-L1 expression)	Comments	Ref
Solid tumors							
NSCLC	221	FISH	5 (11/221)	NR	PD-L1 protein overexpression in all cases with gains	Slight predisposition of CNAs in SCCs	[13]
SCLC	210	qPCR SNP arrays	NR	1.9 (4/210)	High PD-L1 expression in the cases with focal and high-level amplification	Susceptibility of this tumor subset to immune checkpoint blockade	[14]
SCC of vulva and cervix	71	FISH NGS	12.5 (cervical-NGS) 44 (cervical-FISH) 17 (vulvar-FISH)	23 (cervical-FISH) 26 (vulvar -FISH)	Highest PD-L1 expression in co-amplified cases, whereas lowest PD-L1 expression in cases with disomy	Detection of cogain or coamplification in both PD-L1 and PD-L2 genes	[15]
TNBC	183	FISH	8.7 (16/183)	NR	High PD-L1 protein expression in patients with copy number gains	Prolonged disease-specific OS in patients with high PD-L1 basal-like tumors or with gene copy number gains	[16]
BC	1980	aCGH	3.3 (65/1980)	0.25 (5/1980)	High PD-L1 protein expression in the three examined cases with amplification	Classification as IntClust 10 subtype: ✓ Four out of five (80%) cases with amplification ✓ 37/65 (57%) of the tumors with copy number gain	[27]
BC	3145	aCGH	5 (163/3145)	1 (39/3145)	NR	✓ Basal subtype: 74% of the amplified cases and more gains than other subtypes ✓ Losses: 4% (134/3145) ✓ Correlation of gains with elevated PD-L1 mRNA	[28]
TNBC Glioblastomas Colon carcinomas PDAs	41 44 68 150	qPCR FC aCGH	NR	29 (12/41) 4.5 (2/44) 2.9 (2/68)	NR	In TNBC patients with the PDJ amplicon: worse DFS and OS and correlation of amplicon with high mRNA expression of PD-L1 and JAK2	[17]
SCC of the oral cavity	80	FISH	Restriction to tumor cells Absence in the inflammatory cell component	15 (12/80): high-level amplification 4 (3/80): low-level amplification	PD-L1 positivity in 73% of the amplified cases	Mostly HPV-negative SCCs 16/80 (20%) cases with polysomy 49/80 (61%) cases with disomy	[18]
Pulmonary SCCs and ADCs	159	FISH	13.7 (21/159): high gains (mean ≥4) 20.3 (33/159): gains (mean ≥2.5)	8.8 (14/159)	PD-L1 positivity (≥1%) in: 86% (12/14) of amplified cases 29.6% (16/54) of cases with gains	Identification of 9 (5.7%) JAK2 amplified cases, 7 of which with PD-L1 expression ✓ 11/14 (9%) of amplified tumors: ADC ✓ 3/14 (6%): SCC	[19]
Hematological and lymphoid tumors							
DLBCL	190	RNA-seq FISH WGS	12	3	Correlation with elevated PD-L1 expression in cases with cytogenetic changes	Detection of translocations (4%) in PD-L1/PD-L2 locus. Higher frequency of CNAs in the non-GCB subtype	[20]
cHL	108	FISH	56 (61/108)	36 (39/108)	Correlation of genetic alterations with PD-L1 expression (especially in disomic cases)	Correlation of gene amplification with reduced PFS. Higher amplification frequency in patients with advanced stage disease	[21]
HL	10	FISH	60	40	Correlation with PD-L1 increased expression in all cases with CNAs	Association with activation of JAK/STAT3 signaling pathway	[22]
NSHL PMBCL MCHL	16 7 41	qPCR	NR	38 (6/16) 0 63 (26/41)	Association with PD-L1 protein expression in NSHL amplified cases	Association of *JAK2* amplification with elevated PD-L1 transcription Correlation of PMBCL cases with increased PD-L1 transcript	[23]
Primary B-cell NHL	67	Oligonucleotide capture sequencing	NR	NR	Significant association between rearrangements and PDL protein expression	Detection of 36 novel rearrangements (17 inversions/deletions/duplications and 19 translocations)	[24]
PMBCL	125	FISH	26	29	NR	Correlation of genetic alterations with increased PDL transcripts (especially in break-apart positive cases)	[25]
PCNSLs PTLs	50 43	HD-SNP	67/63 (EBV+ /EBV-35) (15/43)	NR	Increased PD-L1 expression in copy number gain(+) cases	Translocations in 6% of EBV- PCNSLs and 4% of PTLs	[26]

*NSCLC* non-small cell lung carcinoma, *SCLC* small-cell lung carcinoma, *SCC* squamous cell carcinoma, *BC* breast cancer, *TNBC* triple-negative breast carcinoma, *PDA* pancreatic ductal adenocarcinomas, *PDJ amplicon* the loci for PD-L1, PD-L2, and JAK2, *DLBCL* diffuse large B-cell lymphoma, *cHL* classical Hodgkin lymphoma, *NSHL* nodular sclerosing Hodgkin lymphoma, *NHL* non-Hodgkin lymphoma, *PMBCL* primary mediastinal B-cell lymphomas, *PCNSLs* primary central nervous system lymphomas, *PTLs* primary testicular lymphomas, *EBV* Epstein–Barr virus, *IHC* immunohistochemistry, *NR* not reported, *OS* overall survival, *PFS* progression-free survival, *DFS* disease-free survival, *non-GCB* non-germinal center B-cell-like cell, *FISH* fluorescent in-situ hybridization, *qPCR* quantitative polymerase chain reaction, *SNP* single-nucleotide polymorphism,*NGS* next-generation sequencing, *FC* flow cytometry, *aCGH* oligonucleotide array-based comparative genomic hybridization, *RNA-seq* RNA-sequencing, *WGS* whole-genome sequencing, *MCHL* mixed cellularity Hodgkin lymphoma, *HD-SNP* high-density single-nucleotide polymorphism arrays, *CN* copy number, *ADC* adenocarcinomas

Furthermore, a novel genetic regulatory mechanism of PD-L1 gene expression involving the disruption of its 3′ untranslated region (3′-UTR) has been shown in multiple tumor types including T-cell leukemia/lymphoma, DLBCL, and gastric adenocarcinoma. Through interruption of PD-L1 3′-UTR by structural variation, a deviant increase in PD-L1 transcripts occurs leading to immune escape in murine EG7-OVA cancer cells, which in turn can be reversed by PD-L1/PD-1 inhibition [30].

## PD-L1 regulation via oncogenic signaling pathways

### RAS/RAF/MEK/MAPK-ERK pathway

The mitogen-activated protein kinase (MAPK) pathway is crucial for various functions in normal cells, including growth and differentiation. Its role is also important in carcinogenesis because its activation leads to cancer development [31]. The ERK-MAPK pathway has been shown to regulate PD-L1 expression in different cancer types. Both pharmacologic inhibition of mitogen-activated protein kinase (MEK) and small interfering RNA (siRNA) knockdown of ERK1/2 resulted in decreased levels of PD-L1 in melanoma cells resistant to BRAF inhibition [32]. Interestingly, in TNBC cells, MEK inhibition resulted in upregulation of MHC II and PD-L1 expression both in vitro and in vivo, whereas combined MEK/PD-1 inhibition increased the effectiveness of antitumor immunity [33]. MAPK signaling pathway was also responsible for the ectopic expression of PD-L1 in v-Ki-ras2 Kirsten rat sarcoma viral oncogene homolog (KRAS)-mutant NSCLC cell lines, as revealed by the decrease in PD-L1 levels after both MEK and extracellular signal-regulated MAP kinase (ERK) abrogation [34]. In another study, Toll-like receptor 4 activation resulted in upregulation of PD-L1 in bladder cancer cells. The use of both ERK and JNK inhibitors abrogated PD-L1 expression, further supporting the contribution of MAPK signaling in PD-L1 regulation [35]. Moreover, the interaction of tyrosine kinase receptor c-Met with its ligand hepatocyte growth factor (HGF) induced Ras activation. Ablation of Ras effect led to downregulation of c-Met-mediated expression of PD-L1 in renal cancer cells [36].

KRAS activation may also induce PD-L1 expression, as it resulted in stabilization of PD-L1 mRNA transcript assessed through Adenylate-uridylate-rich elements identification in its 3′-UTR in lung cancer cell lines. Additionally, MEK and Phosphoinositide 3-kinase (PI3K) inhibition led to decreased PD-L1 levels and enhanced effectiveness of antitumor immunity in vivo [37].

### PI3K/PTEN/Akt/mTOR pathway

The PI3K/Akt/mTOR signaling represents another pathway that affects immune surveillance through the regulation of PD-L1. Its activation by either oncogenic *PIK3CA* mutations (catalytic subunit alpha of PI3K) or by loss-of-function mutations of its negative regulator, phosphatase and tensin homolog (PTEN) modulates immune responses contributing to a survival benefit of cancer cells [38]. In human gliomas, loss of PTEN and activation of PI3K pathway enhanced PD-L1 expression [39]. In TNBC, knockdown of PTEN by short hairpin RNA resulted in elevated levels of both PD-L1 protein expression and mRNA transcripts, whereas inhibition of Akt and mechanistic target of rapamycin (mTOR) decreased PD-L1 expression [40]. In a murine model of lung SCC, concurrent inactivation of PTEN and Lbk1 resulted in increased levels of PD-L1 [41]. PI3K inhibition, resulted in PD-L1 downregulation in different cancer types including renal cell carcinoma through HGF/c-Met [36], *KRAS-* or *EGFR*-mutated NSCLC [42] and melanoma [32]. Conversely, LY294002 did not abrogate PD-L1 expression in bladder cancer cells [35]. Moreover, mTOR inhibition with rapamycin reduced levels of PD-L1, both in human cell lines and in murine models of NSCLC and combined treatment with rapamycin and anti-PD-1 antibody inhibited tumor growth in mice [42].

### Epidermal growth factor receptor (EGFR)

*EGFR* is commonly mutated in NSCLC and has been associated with PD-L1 upregulation in these tumors [43]. PD-L1 was overexpressed in *EGFR*-mutant murine lung cancer, whereas treatment with an anti-PD-1 antibody restrained tumor growth. Forced ectopic expression of mutant *EGFR* on bronchial epithelial cells resulted in PD-L1 upregulation, whereas this effect was abolished upon treatment with EGFR tyrosine kinase inhibitors [44, 45]. The EGFR-mediated regulation of PD-L1 in EGFR mutant NSCLC was dependent on MAPK pathway activation. Inhibition of ERK1/2/c-Jun resulted in reduced PD-L1 levels in PD-L1 overexpressing lung cancer cells [46]. In another more recent study, EGFR was shown to regulate the expression of PD-L1 through the activation of Interleukin-6 (IL-6)/Janus Kinase (JAK)/signal transducer and activator of transcription 3 (STAT3) pathway in EGFR-driven NSCLC [47].

### EML4-ALK

PD-L1 upregulation has been observed in patients with NSCLC harboring the anaplastic lymphoma kinase (ALK) and echinoderm microtubule-associated protein like-4 (EML4) chromosomal rearrangement. Activation of *EML4-ALK* was associated with increased PD-L1 expression; furthermore, treatment with either the ALK inhibitor alectinib or ALK gene silencing with siRNA abrogated this effect. Notably, PD-L1 upregulation was dependent on MAPK/ERK/MEK and PI3K/Akt signaling pathways [48]. In another study using pulmonary adenocarcinoma cell lines, EML4-ALK transcriptionally regulated PD-L1 via STAT3 and HIF-1a [49]. These studies indicate the different ways in which this chimeric protein can regulate the expression of PD-L1 and thus reveal the complexity of signaling pathways and their downstream targets. The various crosstalks in the cellular level can influence anticancer immunity and at the same time offer possible appealing therapeutic targets.

## Transcriptional control of PD-L1

**Fig. 1:**
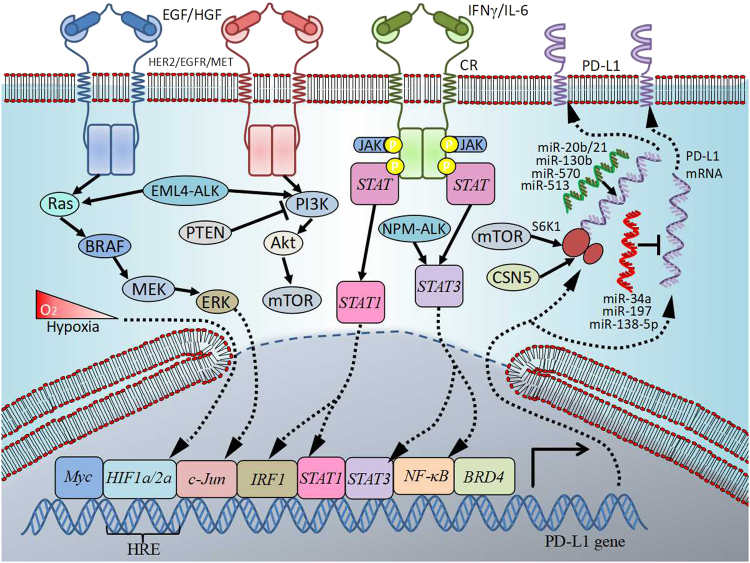
Transcriptional and post-transcriptional control of PD-L1 in cancer. Regulation of PD-L1 is complex and occurs at different levels. Several signaling pathways are involved including RAS/RAF/MEK/MAPK-ERK and PI3K/PTEN/Akt/mTOR. Their activation by oncogenic and/or loss-of-function mutations can lead either to direct action on target genes or to the activation of transcription factors. Such molecules as STAT3, STAT1, c-Jun, HIFs, or NF-κB can shuttle into the nucleus, bind to specific sites on PD-L1 gene promoter and induce its expression. PD-L1 is also regulated post-transcriptionally by microRNAs, which bind to mRNA and lead to its translational repression or enhancement

The transcriptional regulation of PD-L1 is summarized in Fig. 1.

### The JAK/STAT pathway

STAT3 plays a key role in promoting cancer cell survival and proliferation, as well as creating immunosuppressive and thus pro-carcinogenic conditions in the tumor microenvironment (TME) [50]. Furthermore, STAT3 is involved in PD-L1 regulation in various cancer types. In nucleophosmin-anaplastic large-cell lymphoma kinase (NPM-ALK) positive anaplastic large-cell lymphoma (ALCL), STAT3 is activated by NPM-ALK oncoprotein through JAK3 activation, binds physically to the PD-L1 gene promoter, and induces its expression in vitro and in vivo [51]. This STAT3-mediated transcriptional regulation of PD-L1 has been recently shown in another T-cell lymphoma, namely the ALK-negative ALCL. STAT3 gene silencing led to decreased PD-L1 levels in ALK-ALCL [52] and also in *KRAS*-mutant NSCLC cell lines [34]. By contrast, chromatin immunoprecipitation analysis did not show active binding of STAT3 directly on the promoter of *PD-L1* in melanoma cells, despite the presence of putative binding sites of STAT3 on the promoter identified in silico. Abrogation of STAT3 resulted in enhancement of PD-L1 construct activity mediated by IFNg [53]. PD-L1 was also induced by latent membrane protein-1 in Epstein–Barr virus (EBV)-associated nasopharyngeal carcinomas (NPC) through JAK3/STAT3 activation [54].

Another STAT family member, STAT1, is considered to be a tumor suppressor that reduces proliferation, induces apoptosis, and enhances cancer immunosurveillance [55]. Accumulating evidence indicates the emerging role of STAT1 in tumor growth, immune suppression, and therapeutic resistance [56]. Upon stimulation with IFNg, STAT1 activation resulted in PD-L1 upregulation and in reduction of NK-cell activity against tumor cells in multiple myeloma, acute myeloid leukemia (AML), and acute lymphoblastic leukemia (ALL) [57]. Similarly, STAT1 inhibition led to decreased PD-L1 levels in myeloma cells and thus suppressed the antitumor function of cytotoxic T cells [58]. PD-L1 upregulation was JAK2/STAT1-dependent in head and neck cancer with wild-type EGFR, whereas JAK2 inhibition resulted in both basal and EGF-mediated downregulation of PD-L1. Moreover, knockdown of STAT1 gene abolished both IFNg- and EGF-mediated upregulation of PD-L1. Of note, EGFR activation promotes phosphorylation of STAT1, which in turn binds to the promoter of PD-L1 and controls its expression [59]. Although putative binding sites for STAT1 on PD-L1 promoter have been postulated, active binding of STAT1 on PD-L1 gene promoter could not be demonstrated in melanoma cells [53].

Interferon regulatory factor 1 (IRF1) is a downstream effector of STAT1 upon IFNg stimulation. Its role is crucial in both constitutive and IFNg-mediated upregulation of PD-L1. Inhibition of IRF1 activity or expression resulted in decreased PD-L1 levels in human lung cancer cells [60]. The key role of IRF1 and interferon receptor pathway in the regulation of PD-L1 has also been implied in melanoma, where putative binding sites for IRF1 have been identified in the PD-L1 promoter and abrogation of IRF1 site resulted in reduced PD-L1 levels [53, 61]. Recently, another novel mechanism of PD-L1 regulation by DNA double-strand breaks (DSBs) was unveiled. This DSB-dependent PD-L1 upregulation was mediated by the activation of STAT1/STAT3 phosphorylation and IRF1 [62].

### Hypoxia-inducible factors (HIFs)

Hypoxia signaling represents an important pathway in oncogenesis. HIF-1a and HIF-2a are the major components of a transcriptional complex, through which tumor cells adapt to hypoxic conditions. HIF stabilization leads to its binding to specific regions called hypoxia response elements (HRE) on certain gene promoters [63]. High levels of HIF-1 have been correlated with both worse outcomes and resistance to cytotoxic therapy [64]. Intriguingly, HIF-1 expression by different cellular sub-populations of the innate and adaptive immunity can modify antitumor activity by repressing the effective T-cell response and alter TME to promote tumor cell survival [63]. A recent study revealed that HIF-1α guided CD8 + T-cell migration and function, whereas its depletion on T cells resulted in increased tumor growth and impaired antitumor control [65]. One of the mechanisms by which hypoxia signaling impairs T-cell functionality is the induction of PD-L1 on myeloid-derived suppressor cells under hypoxic conditions. Indeed, HIF-1a transcriptionally regulates PD-L1 expression by binding on HRE of its promoter [66]. Furthermore, PD-L1 may be a target of HIF2a in clear cell renal cell carcinoma (ccRCC) cells in which the tumor-suppressor pVHL was abrogated. Upon deficiency of pVHL increased PD-L1 levels, associated with HIF-2a activation, were observed in vitro [67]. Similar results were obtained from ccRCC patient samples with VHL loss-of-function mutations, where a positive correlation was seen between PD-L1 expression, HIF-2a expression and VHL mutations. Of note, HIF-2a transcriptionally regulates PD-L1 by binding to the active HRE of its promoter [68]. Moreover, STAT3 can cooperate with HIF-1, but not HIF-2, in the regulation of HIF target genes in response to hypoxia. Inhibition of STAT3 expression or activity in breast and RCC cell lines reduced the expression of genes targeted by HIF-1 [69]. These findings support the idea of combining HIF-targeting therapies and immunotherapy.

### The role of nuclear factor kappa B (NF-κB)

NF-κB is a master transcription factor activated in several cancer types, promoting inflammation, inhibiting apoptosis, and impairing effective antitumor immunity [70]. The NF-κB family contains seven members, with the most representative being the p65 RelA/p50. This cytoplasmic heterodimer translocates to the nucleus and acts as a transcription factor of κB upon degradation of the IκΒ-α inhibitor [71, 72]. In melanoma cells, NF-κB mediated PD-L1 overexpression induced by IFN-γ. PD-L1 upregulation by NF-κΒ was independent of STAT3 and c-Jun, whereas targeting of MAPK and PI3K signaling pathways had a minor impact on PD-L1 expression [72]. Notably, STAT3 regulates and cooperates with NF-κΒ in additional cancer types [73]. For example, PD-L1 regulation may be dependent on p65/NF-κB and mediated by LMP1 in EBV-positive NPC, as inhibition of NF-κB activity resulted in decreased PD-L1 levels [54].

### The Myc oncogene

*Myc* plays a pivotal role in carcinogenesis by controlling cell proliferation and survival in various cell systems. Tumor regression after Myc inactivation is associated with a not fully understood immune response, as reflected by the accumulation of CD4 + T cells [74–76]. Furthermore, a novel role of *Myc* was recently revealed in the context of avoiding effective cancer immunosurveillance. Using a Tet-off MYC-dependent mouse model of T-ALL (MYC T-ALL), Casey et al. showed that *Myc* transcriptionally regulates PD-L1 and CD47, an inhibitory regulator of the innate immune system [77]. Moreover, forced expression of PD-L1 and CD47 upon Myc inactivation was correlated with worse antitumor immune response as indicated by the reduction of macrophages and CD4 + T cells in TME, tumor progression, and maintenance of angiogenesis and senescence [78]. Elucidating the role of Myc in the regulation of immune-mediated antitumor response, the potential crosstalks with other oncogenic pathways and the immune infiltrate in TME may pave the way for the use of immune checkpoint inhibitors in patients with Myc-overexpressing tumors [79]. A recent work on ALK-negative ALCL also supports a Myc-mediated regulation of PD-L1, as forced expression of *Myc* led to PD-L1 upregulation in cell lines showing low baseline levels of PD-L1. Similarly, both inhibition and silencing of Myc resulted in PD-L1 downregulation in lymphoma cells [52].

### The bromodomain and extraterminal (BET) protein BRD4

BET proteins modulate gene expression through enzymes that regulate chromatin and histone modification [80]. Specifically, the BET protein BRD4 acts through RNA polymerase II by binding to the acetyl-lysine region of histones [81]. Inhibition of BRD4 by the JQ1 inhibitor decreased PD-L1 expression and tumor growth. BRD4 gene silencing also resulted in decreased PD-L1 levels in mouse models and in ovarian cancer cell lines. Notably, BRD4 transcriptionally regulated PD-L1 by binding on its promoter [82]. Similarly, in a recent study on B-cell lymphoma, BET inhibitors enhanced effective antitumor immunity through regulation of PD-L1, whereas inhibition and genetic ablation of BRD4 resulted in suppression of PD-L1 expression in a transcriptional, Myc-independent, manner. Moreover, BRD4 synergized with IRF1 to regulate PD-L1 expression induced by IFN-γ [83]. Also, another BET inhibitor (I-BET151) was shown to abrogate NF-Κβ activity in melanoma, both in vitro and in vivo, thus indirectly affecting PD-L1 expression [84].

### Histone deacetylases (HDACs)

The role of the epigenetic modifiers HDACs in the modification of non-histone targets, including those participating in tumor-host interactions, has recently been investigated [85, 86]. In a study in melanoma, both inhibition and depletion of HDAC6 resulted in reduced PD-L1 levels in vitro and in vivo. PD-L1 regulation by HDAC6 was mediated by STAT3 and both HDAC6 and STAT3 were recruited to the PD-L1 gene promoter [87]. It should be noted that HDAC have pleiotropic effects within both the innate and adaptive immune response, and may thus affect PD-L1 levels via interferons [88].

### The role of cell cycle

Cyclin-dependent kinases (CDKs) have a key role in cell cycle [89]. Cyclin-dependent kinase 5 (Cdk5) is a serine-threonine kinase important in central nervous system function [90] and other cellular functions [91, 92]. In a study of medulloblastoma, depletion of Cdk5 led to the upregulation of interferon regulatory factor 2 and interferon regulatory factor binding protein 2, which in turn, suppressed the expression of PD-L1. Cdk5 was thus necessary for PD-L1 upregulation after IFN-γ stimulation through STA1/IRF1 axis and its disruption led to tumor rejection in a CD4 + T-cell-dependent manner in medulloblastoma mouse models [93]. These data highlight Cdk5 as a novel target for interventions in combination with immune checkpoint blockade. Additionally, CDK4/6 inhibition has been recently shown to enhance antitumor immunity through increased T-cell cytotoxicity and Treg suppression [94]. This is discussed in detail in the post-translational regulation of PD-L1 hereunder.

### The AP-1 transcription factors

c-Jun, the best known member of the AP-1 family, represents another transcription factor that is implicated in PD-L1 gene regulation. Knockdown of c-Jun resulted in decreased levels of PD-L1 in melanoma cells resistant to BRAF inhibitors [32], and co-activation of STAT3 and the subsequent formation of a transcriptional complex further enhanced these effects [95]. Similarly, combined knockdown of c-Jun and STAT3 genes in the same melanoma model showed a synergistic effect on PD-L1 downregulation [32]. Additionally, c-Jun and JUNB have been shown to bind AP-1 sites in the PD-L1 promoter in HL cells [96] and in KRAS-mutant NSCLC. In lung adenocarcinoma cell lines, the transcriptional activity was subjected to MAPK signaling pathway [34]. MAPK/AP-1 was also shown to contribute to LMP1-mediated upregulation of PD-L1 in EBV-associated NPC [54].

### The ambivalent role of p53

The tumor-suppressor gene p53 has been implicated in antitumor immunity by regulating several genes involved in the immune system. Indeed, immune checkpoint regulation has been shown to represent a major target of p53 [97]. Paradoxically, activation of wild-type p53 using the small molecule Nutlin-3a resulted in increased expression of PD-L1 in human breast cancer [98] and in ALK-negative ALCL cells [52]. In p53-mutated NSCLC, downregulation of miR-34 resulted in increased PD-L1 levels [99], whereas an inverse correlation between miR-34a and PD-L1 was also confirmed in AML [100].

### MicroRNAs

MicroRNAs can bind to 3′-UTR of mRNAs and lead to their degradation or translational repression [101]. MiR-513 was shown to increase PD-L1 expression in cholangiocytes [102], whereas mutation in the 3′-UTR of PD-L1 mRNA led to overexpression of the protein by preventing miR-570 binding in gastric cancer [103, 104]. On the contrary, miR-197 downregulated PD-L1 by affecting STAT3 in platinum-resistant NSCLC [105], whereas miR-138-5p was associated with decreased levels of PD-L1 in colorectal cancer (CRC) [106]. Also in CRC, miR-20b, miR-21, and miR-130b caused PD-L1 upregulation through attenuation of PTEN [107].

## Post-translational regulation of PD-L1

### The role of ubiquitination

In a recent study by Lim et al., a novel regulatory mechanism involving the fifth protein element of COP9 signalosome complex (CSN5), also known as Jab1, was revealed in breast cancer. CSN5 has been associated with increased proliferation, decreased apoptotic rates, and survival of cancer cells [108]. Under chronic inflammatory conditions, tumor necrosis factor alpha (TNF-α), secreted mostly by macrophages, led to PD-L1 stabilization and therefore to an immunosuppressive profile of the tumor environment [61]. The stabilization of PD-L1 by TNF-a was shown to be mediated by NF-κΒ subunit RelA/p65, which binds on the promoter of CSN5 gene and has a direct effect on its regulation. CSN5 in turn, prevents the ubiquitination of PD-L1, hinders its degradation and as a result enhances tumor escape from immunosurveillance. Indeed, CSN5 inhibition or gene silencing abolished PD-L1 expression and tumor proliferation in vivo. Curcumin, a CSN5 inhibitor, induced better responses to anti-CTLA-4 treatment in vitro, indicating the potential of combinational administration of immune checkpoint with CSN5 inhibitors [61, 109, 110]. In another in vitro study, induction of both PD-L1 ubiquitination and PD-L1 protein levels was noted upon treatment with epidermal growth factor. An increase of mono- and multiubiquitination of PD-L1 was seen, an effect that was abrogated upon inhibition of the EGFR pathway and/or ubiquitin E1 activating enzyme [111]. Furthermore, a recent study demonstrated a novel role of cyclin D-CDK4 and cullin 3-speckle-type POZ protein (SPOP) E3 ligase in regulating the expression of PD-L1. Cyclin D1-CDK4 was shown to phosphorylate SPOP and lead to ubiquitination-mediated PD-L1 destabilization. Thus, either inhibition of CDK4/6 or loss-of-function mutations of SPOP led to increased levels of PD-L1 and reduced tumor-infiltrating lymphocytes. Additionally, treatment with a CDK4/6 inhibitor and an anti-PD-1 antibody resulted in tumor regression and improved survival in vivo [112].

### Lysosomal-mediated degradation

CKLF-like MARVEL transmembrane domain containing protein 6 (CMTM6) was recently identified as a novel regulator of PD-L1 [113, 114]. CMTM6—a tetraspanin protein—interacted with PD-L1 through its transmembrane domain and regulated PD-L1 expression in cancer and myeloid cells, both in vitro and in vivo [115]. Depletion of CMTM6 did not influence the *CD274* transcript, but led to reduction of PD-L1 protein expression and augmentation of antitumor immunity. The mechanism of action of CMTM6 involves the avoidance of PD-L1 lysosome-mediated degradation, probably through prevention of its ubiquitination, as these two proteins are co-localized in the plasma membrane [116].

### The role of glycosylation

*N*-glycosylation represents a crucial post-translational modification determining protein formation, functionality, and interaction with other proteins [117]. A novel association between procedure-glycosylation and ubiquitination in the regulation of PD-L1 has recently been unveiled. In basal-like breast cancer cells, *N*-glycosylation of PD-L1 (highly at sites N35, N192, N200, and N219) led to protein stabilization and avoidance of its degradation by 26S proteasome. In contrast, non-glycosylated forms interrelated with Glycogen synthase kinase 3 beta (GSK3β), which in turn phosphorylated PD-L1 resulting in its degradation. Inhibition of GSK3β activity augmented immune suppression by tumor cells both in vitro and in vivo. Furthermore, EGFR promoted inactivation of GSK3β, and EGFR signaling blockade reversed stabilization of PD-L1 and led to enhanced antitumor responses [118]. In another study, N-linked glycosylation of PD-L1 (gPD-L1) was shown to increase PD-L1/PD-1 interaction, and consequently immunosuppression in TNBC. Its targeting with monoclonal antibodies or drug-conjugated gPD-L1 was thus proposed as a promising target of post-translational modifications of immune checkpoints [119].

## Effect of chemotherapy in PD-L1 expression

Chemotherapeutic agents, apart from their direct cytotoxic effects on cancer cells, can also modulate immune responses against tumors [120, 121]. Treatment with paclitaxel, etoposide and 5-fluorouracil induced PD-L1 expression in breast cancer cell lines in a dose-dependent manner [122]. Paclitaxel was also associated with elevated levels of PD-L1 in human CRC and hepatocellular carcinoma cell lines. This regulation was dependent on MAPK activation [123]. Likewise, cisplatin induced PD-L1 expression in hepatoma cells in ERK1/2 phosphorylation-dependent manner [124]. In another study, doxorubicin led to PD-L1 downregulation on cell surface and a simultaneous PD-L1 upregulation in the nucleus of breast cancer cells. Nuclear PD-L1 expression was accompanied by nuclear AKT phosphorylation and proved to be dependent on PI3K/AKT pathway, whereas knockdown of PD-L1 was associated with enhanced doxorubicin-mediated apoptosis [125].

## Targeting immune checkpoint regulators: the era of immunotherapy in cancer

The introduction of systemic cancer immunotherapy in clinical practice significantly predates the first randomized trials of immune checkpoint inhibitors. Despite the occurrence of rare, prolonged complete remissions in patients with metastatic melanoma and ccRCC [126, 127], the use of high-dose IL-2 was restricted by the significant, often fatal adverse events and the need for intensive monitoring and experience in its administration, whereas the use of IFNg in ccRCC was characterized by its perceived low efficacy [128]. The clinical application of cancer immunotherapy had remained stagnant until the first checkpoint inhibitor received regulatory approval for use in metastatic melanoma, the CTLA-4 inhibitor ipilimumab. Ipilimumab exhibits several recurring characteristics of immunotherapy: slow induction of response, a striking disassociation between imaging-assessed objective responses and survival, which led to the introduction of immune-related response criteria [129], unique patterns of toxicity termed “immune-related adverse events” [130] and robust, durable improvements in terms of patient survival [131].

Shortly after the approval of ipilimumab the first trials of PD-1 and later PD-L1 inhibitors were published. Their results have vastly changed the treatment landscape in multiple human malignancies, adding a new category of effective and, compared with cytotoxic chemotherapy, less toxic agents to the therapeutic armamentarium. The results of the published phase 3 trials are presented in Table 2 [132–148], whereas a selection of ongoing randomized trials in an ever-expanding list of indications, both at refractory disease, as well as in earlier lines of therapy or at the adjuvant setting is presented in Table 3. The results of these trials are eagerly awaited, because there are high unmet needs in many of the indications that these agents are being tested. Of interest are also hematologic malignancies; preliminary trials report impressive response rates in otherwise refractory disease [149], believed to be driven by both the inherent role of the PD-1/PD-L1 axis in the evasion of immunosurveillance in lymphoid tumors, particularly in those with a viral etiology [150], and by the presumed significance of PDL1 and PDL2 amplification in the biology of certain neoplasms such as Hodgkin lymphoma [22]. In contrast, the recent discontinuation of the ongoing phase 3 trials in multiple myeloma due to an increased risk of death underscores the fact that better understanding of the underlying immune mechanisms is still needed.

**Table 2:** Randomized phase 3 trials of PD-1 and PD-L1 inhibitors

Trial [Ref]	*N*	Clinical setting	Comparison	ORR (%)	PFS (months)	OS (months)
Non-small cell lung cancer						
KEYNOTE-024 [132]	305	First line	Pembrolizumab vs platinum doublet	44.8 vs 27.8	10.3 vs 6.0, *p* < 0.001	HR = 0.60 (0.41–0.89), *p* = 0.005
CheckMate 026 [133]	541	First line	Nivolumab vs platinum doublet	26 vs 33 (NS)	4.2 vs 5.9, *p* = 0.25	14.4 vs 13.2 (NS)
KEYNOTE-010 [134]	1034	Second line	Pembrolizumab (2 schedules) vs docetaxel	18 and 18 vs 9, *p* = 0.0005 and *p* = 0.0002	3.9 and 4.0 vs 4.0 (NS)	10.4 and 12.7 vs 8.5, *p* = 0.0008 and *p* < 0.0001
CheckMate 017 [135]	272	Second line, squamous	Nivolumab vs docetaxel	20 vs 9, *p* = 0.008	3.5 vs 2.8, *p* < 0.001	9.2 vs 7.3, *p* < 0.001
CheckMate 057 [136]	582	Second line, non-squamous	Nivolumab vs docetaxel	19 vs 12, *p* = 0.02	2.3 vs 4.2, *p* = 0.39	12.2 vs 9.4, *p* = 0.002
OAK [137]	850	Second line	Atezolizumab vs docetaxel	14 vs 13 (NS)	2.8 vs 4.0, *p* = 0.49	13.9 vs 9.6, *p* = 0.0003
PACIFIC [145]	713	Maintenance stage III	Durvalumab vs placebo	28.4 vs 16.0, *p* < 0.001	16.8 vs 5.6, *p* < 0.001	Not reported
Cutaneous melanoma						
KEYNOTE-006 [138]	834	First line	Pembrolizumab (2 schedules) vs ipilimumab	33.7 and 32.9 vs 11.9, *p* < 0.001	HR = 0.58 (0.46–0.72), p < 0.001	HR = 0.63 (0.47–0.83), p = 0.0005
CheckMate 066 [139]	418	First line	Nivolumab vs dacarbazine	40.0 vs 13.9, *p* < 0.001	5.1 vs 2.2, *p* < 0.001	HR = 0.42 (33.0–50.9), *p* < 0.001
CheckMate 037 [140]	405	After ipilimumab	Nivolumab vs dacarbazine or carboplatin/paclitaxel	31.7 vs 10.6	3.1 vs 3.7 (NS)	16 vs 14 (NS)
CheckMate 067 [141]	945	First line	Nivolumab + ipilimumab vs nivolumab vs ipilimumab	58 vs 44 vs 19	11.5 vs 6.9 vs 2.9	NR vs NR vs 20
CheckMate 238 [146]	906	Adjuvant	Nivolumab vs ipilimumab	HR for RFS 0.65 (97.56% CI, 0.51–0.83), *p* < 0.001		
Urothelial bladder cancer						
KEYNOTE-045 [142]	542	Second line	Pembrolizumab vs paclitaxel or docetaxel or vinflunine	21.1 vs 11.4, *p* = 0.001	2.1 vs 3.3, *p* = 0.42	10.3 vs 7.4, *p* = 0.002
Imvigor 211^a^ [147]	931	Second line	Atezolizumab vs paclitaxel or docetaxel or vinflunine	23.0 vs 21.6 (NS)	2.4 vs 4.2 (NS)	11.1 vs 10.6, *p* = 0.41
Clear cell renal carcinoma						
CheckMate 025 [143]	821	After 1–2 TKIs	Nivolumab vs everolimus	25 vs 5, *p* < 0.001	4.6 vs 4.4, p = 0.11	25.0 vs 21.8, *p* ≤ 0.0148
CheckMate 214^b^ [148]	1096	First line	Nivolumab + ipilimumab vs sunitinib	42 vs 27, *p* < 0.0001	22.6 vs 8.4, *p* = 0.0331	NR vs 32, *p* = 0.0003
Head and neck squamous cell carcinoma						
CheckMate 141 [144]	361		Nivolumab vs methotrexate or docetaxel or cetuximab	13.3 vs 5.8	2.0 vs 2.3, *p* = 0.32	7.5 vs 5.1, *p* = 0.01

*ORR* objective response rate, *PFS* progression-free survival, *OS* overall survival, *NS* nonsignificant, *NR* not reached, *HR* hazard ratio, *RFS* relapse-free survival, *CI* confidence interval, *TKI* tyrosine kinase inhibitor

^a^ The results presented here concern the primary endpoint of the study in the IC2/3 group of PD-L1 expression

^b^ The results presented here concern the primary endpoint of the study in the intermediate and poor risk group

**Table 3:** Selected ongoing phase 3 trials of PD-1 and PD-L1 inhibitors

Disease	Trial	Clinical setting	Clinicaltrials.gov Identifier
Pembrolizumab			
Breast cancer			
TNBC	KEYNOTE-119	Prior anthracycline/taxane, vs monochemotherapy	NCT02555657
TNBC	KEYNOTE-522	First line, chemotherapy ± pembrolizumab	NCT03036488
TNBC		Adjuvant in residual disease after neoadjuvant chemotherapy	NCT02954874
HER2 + breast cancer		First line, Paclitaxel/Trastuzumab/Pertuzumab ± pembrolizumab	NCT03199885
Gastrointestinal cancer			
Hepatocellular cancer	KEYNOTE-394	Pretreated (sorafenib or oxaliplatin), vs placebo	NCT03062358
Hepatocellular cancer	KEYNOTE-240	Prior sorafenib, vs placebo	NCT02702401
Gastric cancer	KEYNOTE-063	Second line, vs paclitaxel	NCT03019588
Esophageal cancer	KEYNOTE-590	First line, cisplatin/5FU ± pembrolizumab	NCT03189719
Esophageal cancer	KEYNOTE-181	Second line, vs taxane or irinotecan	NCT02564263
Colorectal cancer	KEYNOTE-177	First line, microsatellite instability-high or mismatch repair deficient, chemotherapy vs pembrolizumab	NCT02563002
Genitourinary cancer			
Renal cell carcinoma	KEYNOTE-564	Adjuvant, vs placebo	NCT03142334
Renal cell carcinoma	KEYNOTE-426	First line, pembrolizumab/axitinib vs sunitinib	NCT02853331
Bladder cancer	KEYNOTE-361	First line, chemotherapy vs pembrolizumab vs combination	NCT02853305
Lung and head and neck cancer			
NSCLC	KEYNOTE-091	Adjuvant, vs placebo	NCT02504372
NSCLC	KEYNOTE-407	First line, squamous cell, chemotherapy ± pembrolizumab	NCT02775435
NSCLC	KEYNOTE-189	First line, non-squamous cell, chemotherapy ± pembrolizumab	NCT02578680
SCLC	KEYNOTE-604	First line, chemotherapy ± pembrolizumab	NCT03066778
Mesothelioma	PROMISE-Meso	Second line, vs gemcitabine or vinorelbine	NCT02991482
Head and neck cancer	KEYNOTE-412	After chemoradiation, vs placebo	NCT03040999
Head and neck cancer	KEYNOTE-048	Chemotherapy vs pembrolizumab vs combination	NCT02358031
Melanoma			
Melanoma	KEYNOTE-252	First line, pembrolizumab ± epacadostat	NCT02752074
Melanoma		Adjuvant, pembrolizumab vs ipilimumab vs interferon alfa-2B	NCT02506153
Hematologic malignancies			
Hodgkin’s lymphoma	KEYNOTE-204	Relapsed/refractory disease, vs brentuximab vedotin	NCT02684292
Multiple myeloma	KEYNOTE-183	Relapsed/refractory disease, pomalidomide/dexamethasone ± pembrolizumab	NCT02576977
Multiple myeloma	KEYNOTE-185	First line, lenalidomide/dexamethasone ± pembrolizumab	NCT02579863
Nivolumab			
Gastrointestinal cancer			
Hepatocellular cancer		First line, vs sorafenib	NCT02576509
Gastric cancer	CheckMate 649	First line, nivolumab/ipiliumab vs nivolumab/chemotherapy vs chemotherapy	NCT02872116
Esophageal and junction cancer	CheckMate 577	Adjuvant, vs placebo	NCT02743494
Esophageal cancer	CheckMate 648	First line, nivolumab/ipilimumab vs nivolumab/chemotherapy vs chemotherapy	NCT03143153
Esophageal cancer		Second line, vs taxane	NCT02569242
Genitourinary cancer			
Bladder cancer	CheckMate 274	Adjuvant, vs placebo	NCT02632409
Bladder cancer	CheckMate 901	First line, nivolumab/ipilimumab vs chemotherapy	NCT03036098
Renal cell carcinoma	CheckMate 9ER	First line, nivolumab/ipilimumab vs nivolumab/cabozantinib vs sunitinib	NCT03141177
Lung and head and neck cancer			
NSCLC	ANVIL	Adjuvant, vs placebo	NCT02595944
NSCLC	CheckMate 816	Neoadjuvant, nivolumab/ipilimumab vs chemotherapy	NCT02998528
NSCLC		Stage III, after chemoradiation vs placebo	NCT02768558
NSCLC	CheckMate 227	First line, nivolumab/ipilimumab vs nivolumab vs nivolumab/chemotherapy vs chemotherapy	NCT02477826
SCLC	CheckMate 451	Maintenance after first line, nivolumab/ipilimumab vs nivolumab vs placebo	NCT02538666
Mesothelioma	CheckMate 743	First line, nivolumab/ipilimumab vs chemotherapy	NCT02899299
Mesothelioma	CONFIRM	Pretreated, vs placebo	NCT03063450
Head and neck cancer	CheckMate 651	First line, nivolumab/ipilimumab vs chemotherapy	NCT02741570
Melanoma			
Melanoma	CheckMate 915	Adjuvant, nivolumab/ipilimumab vs nivolumab vs ipilimumab	NCT03068455
Melanoma		First line BRAF V600E, dabrafenib/trametinib → nivolumab/ipilimumab vs nivolumab/ipilimumab → dabrafenib/trametinib	NCT02224781
Hematologic malignancies			
Hodgkin’s lymphoma	CheckMate 812	Relapsed/refractory disease, nivolumab/brentuximab vedotin vs brentuximab vedotin	NCT03138499
Multiple myeloma	CheckMate 602	Relapsed/refractory disease, pomalidomide/dexamethasone ± nivolumab vs nivolumab/pomalidomide/elotuzumab/ dexamethasone	NCT02726581
Other tumors			
Glioblastoma	CheckMate 143	Second line, nivolumab/ipilimumab vs nivolumab vs bevacizumab	NCT02017717
Glioblastoma	CheckMate 498	First line, radiation and temozolomide or nivolumab	NCT02617589
Atezolizumab			
Breast cancer			
TNBC	IMpassion 031	Neoadjuvant, chemotherapy ± atezolizumab	NCT03197935
TNBC	IMpassion 130	First line, nab-paclitaxel ± atezolizumab	NCT02425891
TNBC	IMpassion 131	First line, paclitaxel ± atezolizumab	NCT03125902
Gastrointestinal cancer			
Colorectal cancer		Pretreated, atezolizumab/cobimetinib vs atezolizumab vs regorafenib	NCT02788279
Colorectal cancer		Adjuvant, microsatellite instability-high or mismatch repair deficient, chemotherapy ± atezolizumab	NCT02912559
Colorectal cancer		First line, microsatellite instability-high or mismatch repair deficient, chemotherapy/bevacizumab ± atezolizumab	NCT02997228
Genitourinary cancer			
Bladder cancer	IMvigor 010	Adjuvant, vs placebo	NCT02450331
Renal cell carcinoma	IMmotion 010	Adjuvant, vs placebo	NCT03024996
Renal cell carcinoma	IMmotion 151	First line, atezolizumab/bevacizumab vs sunitinib	NCT02420821
Prostate cancer	IMbassador 250	Castration-resistant, after anti-androgen and taxane, enzalutamide ± atezolizumab	NCT03016312
Ovarian cancer	ATALANTE	Relapsed, chemotherapy/bevacizumab vs atezolizumab/bevacizumab	NCT02891824
Ovarian cancer	IMagyn 050	First line, Paclitaxel/Carboplatin/Bevacizumab ± atezolizumab	NCT03038100
Lung and head and neck cancer			
NSCLC	IMpower 130	First line, non-squamous, chemotherapy ± atezolizumab	NCT02367781
NSCLC	IMpower 131	First line, squamous, chemotherapy ± atezolizumab	NCT02409355
NSCLC		First line, platinum ineligible, vs monochemotherapy	NCT03191786
SCLC	IMpower 133	First line, chemotherapy ± atezolizumab	NCT02763579
Melanoma			
Melanoma		First line BRAF V600E, vemurafenib/cobimetinib ± atezolizumab	NCT02908672
Durvalumab			
Genitourinary cancer			
Bladder cancer		First line, durvalumab/tremelimumab vs durvalumab vs chemotherapy	NCT02516241
Lung and head and neck cancer			
NSCLC	MYSTIC	First line, durvalumab/tremelimumab vs durvalumab vs chemotherapy	NCT02453282
NSCLC	NEPTUNE	First line, durvalumab/tremelimumab vs chemotherapy	NCT02542293
NSCLC	CAURAL	Second line, EGFR T790M + , osimertinib ± durvalumab	NCT02454933
NSCLC		Adjuvant, vs placebo	NCT02273375
SCLC	Caspian	First line, durvalumab/tremelimumab/chemotherapy vs durvalumab/chemotherapy vs chemotherapy	NCT03043872
Head and neck cancer	KESTREL	First line, durvalumab/tremelimumab vs durvalumab vs chemotherapy	NCT02551159
Avelumab			
Breast cancer			
TNBC	A-Brave	Adjuvant, vs placebo	NCT02926196
Gastrointestinal cancer			
Gastric cancer	JAVELIN Gastric 100	Maintenance after first line, vs continuation chemotherapy	NCT02625610
Gastric cancer	JAVELIN Gastric 300	Third line, vs irinotecan or paclitaxel	NCT02625623
Genitourinary cancer			
Bladder cancer	JAVELIN Bladder 100	Maintenance after first line, vs placebo	NCT02603432
Renal cell carcinoma	JAVELIN Renal 101	First line, avelumab/axitinib vs sunitinib	NCT02684006
Ovarian cancer	JAVELIN Ovarian 100	First line, chemotherapy vs chemotherapy/avelumab vs chemotherapy with avelumab maintenance only	NCT02718417
Ovarian cancer	JAVELIN Ovarian 200	Platinum-resistant relapse, liposomal doxorubicin ± avelumab	NCT02580058
Lung and head and neck cancer			
NSCLC	JAVELIN Lung 100	First line, vs chemotherapy	NCT02576574
NSCLC	JAVELIN Lung 200	Second line, vs docetaxel	NCT02395172
Head and neck cancer	JAVELIN Head and neck 100	Chemoradiotherapy ± avelumab	NCT02952586
Head and neck cancer	REACH	Chemoradiotherapy vs radiotherapy/cetuximab/avelumab	NCT02999087

*NSCLC* non-small cell lung cancer, *SCLC* small cell lung cancer, *TNBC* triple-negative breast cancer, *HER2* human epidermal growth factor receptor 2

Importantly, a new generation of clinical trials has been initiated and initial results are already available regarding a multi-faceted attempt to improve upon the efficacy of PD-1/PD-L1 inhibitors as monotherapy: their combination with CTLA-4 inhibitors, already shown to improve outcomes in metastatic melanoma [141] and pursued in other malignancies including NSCLC and SCLC; their combination with cytotoxic chemotherapy, based upon the premise of the prevention of early disease progression due to the simultaneous administration of chemotherapy and the release of neoantigens due to the cytotoxic effects of the combinatory treatment, which may potentiate the activity of PD-1 inhibitors, an approach that has shown promising results in advanced NSCLC and at the neoadjuvant setting of TNBC [151, 152]; the combination of targeted agents and PD-1 axis blockade [153], with preliminary results showing that combining immunotherapy with inhibitors of known effectors of the axis, such as CDK4/6, results in promising activity [154]; and finally, the combination with inhibitors or stimulators of modulatory molecules such as indoleamine 2,3-dioxygenase (IDO) inhibitors, because IDO is a major negative feedback pathway regulated by IFNg. Preliminary results of the IDO inhibitor epacadostat with nivolumab in a variety of tumors and with pembrolizumab in melanoma are promising and phase 3 results are eagerly awaited [155, 156].

In short, the current era of cancer immunotherapy could be characterized as the “end of the beginning”. A variety of agents is available for use in multiple indications and clinical experience is accumulating. The next phase, namely the optimization of the use of the available agents and the exploration for novel combinations, has already begun.

## Immune checkpoint regulators as novel biomarkers: prognostic and predictive value

Taking into account the significant clinical efficacy of PD-1/PD-L1 blockade in a small subset of patients, the considerable costs and potential for devastating immune-related adverse events associated with the use of these inhibitors and the robust theoretical background explaining the biology of their mechanism of action, considerable efforts have been undertaken in order to identify putative predictive biomarkers. The best characterized biomarker is the immunohistochemistry (IHC)-assessed PD-L1 expression. The conflicting results of individual trials have been summarized in meta-analyses, which indicate that increased levels of PD-L1 expression are associated with an improved probability for objective response [157, 158]. Supporting these results are two recently published clinical trials in the first line of advanced NSCLC, KEYNOTE-024, and CheckMate 026. In the former, overall survival (OS) in patients selected for PD-L1 positivity ≥50% was improved with pembrolizumab compared with platinum-based chemotherapy [132]. Contrary, in the latter trial there were no OS gains in PD-L1 ≥5% patients treated with nivolumab versus chemotherapy [133]. As there are no perceived differences in the potency of these antibodies, the obvious discrepancy in the patient population could account for the different outcome. However, several observations hinder the routine selection of appropriate candidates according to PD-L1 expression. First, in addition to the modest concordance rates between the various antibodies used to assess PD-L1 expression reported in the literature, questions still remain regarding the uncontrolled pre-analytical conditions and the assay and inter-pathologist discrepancies [159], which can lead to PD-L1 status misclassifications despite the similar analytical performance of the available assays [160]. Second, PD-L1 expression exhibits significant intratumoral, intertumoral and temporal heterogeneity [161, 162], putting into question the widespread practice of assessing PD-L1 IHC expression on archival tissue. Third, as clearly shown in individual randomized trials such as the CheckMate 017 trial at the second line of lung SCC [135], characterizing patients as appropriate for anti-PD-1 therapy according to PD-L1 expression both includes patients who do not respond to treatment and also excludes potential responders. Fourth, in the aforementioned CheckMate 026 trial, nivolumab was not more effective than chemotherapy even in the subgroup of 50% or higher PD-L1 expression. As this was not a stratification factor, imbalances such as the sex of the patients could have confounded the results, implying that PD-L1 positivity by itself is not a strong predictive biomarker [133]. Finally, the association of objective response rates and PD-L1 expression in the trial-level meta-analyses is of unsure clinical importance, since checkpoint inhibitors can confer prolonged, clinically meaningful periods of disease stabilization and because their use beyond progression in patients deemed to derive clinical benefit has been found to improve outcomes in a diverse selection of solid malignancies [163–165].

Keeping in mind the shortcomings of PD-L1 expression, other biomarkers have been explored. Following the observation that smokers with NSCLC seem to derive improved benefit from anti-PD-1 agents [166], it was postulated that this effect may be a surrogate marker for an increased mutational load and subsequent increased neoantigen production and exposure and more effective immune response in patients chronically exposed to a strong mutagenic factor such as smoking. Indeed, mutational load has been found to be a predictive factor in NSCLC [167]. Supporting this association is the observation that mismatch repair defective, and thus hypermutated tumors, are exquisitely sensitive to PD-1 blockade [168, 169]. In addition, NSCLC harboring driver molecular aberrations such as *EGFR* mutations, which exhibit lesser mutational loads have been shown to be relatively resistant to immune checkpoint inhibition [170], a finding supported by a recently published meta-analysis on the prediction of response in NSCLC patients. *EGFR* mutant and *KRAS* wild-type status were associated with a lack of sensitivity to PD-1/PD-L1 inhibition, whereas clinical factors such as smoking status, histology, sex, performance status, and age did not affect the magnitude of benefit [171].

The quantitative and qualitative assessment of the host immune response has also been explored as a predictor in checkpoint inhibition. Factors such as the abundance of pre-existing CD8 (+) T cells, a restricted (clonal) TCR repertoire, a TH1-type response, increased levels of IFN-γ and IL-18 and decreased levels of IL-6, among others, have been correlated with improved responses [166, 172], but these results need to be evaluated prospectively in randomized trials. The implementation of multiparametric, high-throughput flow cytometry, and multiplex immunohistochemical staining techniques that vastly improve the T-cell population analysis [173] and of whole-exome sequencing for the evaluation of the mutational load and the presence of specific, predictive molecular alterations will aid in this respect.

On the other hand, PD-1 and PD-L1 expression both at the tissue level and on circulating tumor cells have been evaluated in a wide variety of malignancies for their prognostic impact (Table 4) [17, 21, 174–229]. The results have been thus far inconsistent among tumor types and somewhat confusing, with reports supporting both an improved and a decreased OS conferred by high expression, a phenomenon that resonates the previously mentioned shortcomings of the assessment of PD-L1. The biologic background of these observations is as of yet uncertain. Moreover, as the expansion of the indications of PD-1/PD-L1 blockade continues with the conduct and report of clinical trials, these associations could be affected due to the increasing use of these agents, making their clinical utility questionable at the moment.

**Table 4:** Examples of studies reporting a correlation of PD-1/L1 status and prognosis

Tumor type	PD-1/L1 status	Correlation with outcome	Reference
Breast cancer			
All	↑ PD-L1 expression	Unfavorable	[174, 178, 219]
All	↑ PD-L1 expression	Favorable	[175]
HER2+	↑ PD-L1 expression	Unfavorable	[179]
TNBC	↑ PD-L1 expression	Favorable	[176]
TNBC	PD-L1 amplification	Unfavorable	[16]
Residual after neoadjuvant	↑ PD-L1 expression	Unfavorable	[177]
Gastrointestinal cancer			
All digestive tumors	↑ PD-L1 expression	Unfavorable	[183]
Hepatocellular cancer	↑ PD-L1/2 expression	Unfavorable	[180, 181]
Colorectal cancer	↑ PD-L1 expression	Favorable	[186, 209]
Colorectal cancer	↑ PD-L2 expression	Unfavorable	[187]
Gastric cancer	↑ PD-L1 expression	Unfavorable	[184, 185]
Cholangiocarcinoma	↑ PD-L1 expression	Unfavorable	[217]
Esophageal cancer	↑ PD-L1 expression	Favorable	[214]
Pancreatic cancer	↑ PD-1 expression	Favorable	[182]
Genitourinary cancer			
Clear cell renal	↑ PD-L1/2 expression	Unfavorable	[195–197]
Non-clear cell renal	↑ PD-L1 expression	Unfavorable	[194]
Papillary renal	↑ PD-L1 expression	Unfavorable	[193]
Chromophobe renal	↑ PD-L2 expression	Unfavorable	[192]
Bladder cancer	↑ PD-L1 expression	Unfavorable	[191, 218]
Prostate cancer	↑ PD-1 expression	Unfavorable	[190]
Prostate cancer	↑ PD-L1 expression	Unfavorable	[189]
Ovarian cancer	↑ PD-L1 expression	Favorable	[188, 210]
Lung and head and neck cancer			
NSCLC	↑ PD-L1 expression	Favorable	[211, 213]
NSCLC	↑ PD-L1 expression	Unfavorable	[202–206]
NSCLC	↑ PD-L1 expression	Not predictive	[202]
NSCLC	PD-L1 amplification	Unfavorable	[200]
SCLC	↑ PD-L1 expression	Unfavorable	[201]
Pulmonary neuroendocrine	↑ PD-L1 expression	Unfavorable	[220]
Head and neck cancer	↑ PD-L1 expression	Favorable	[199, 215]
Head and neck cancer	↑ PD-L1 expression	Unfavorable	[198]
Melanoma and sarcoma			
Melanoma	↑ PD-L1 expression	Favorable	[212]
Melanoma	↑ PD-L1 expression	Unfavorable	[208]
Soft tissue sarcoma	↑ PD-L1 expression	Unfavorable	[207]
Hematologic malignancies			
Hodgkin’s lymphoma	↑ PD-1 expression	Unfavorable	[222]
Hodgkin’s lymphoma	PD-1/L-1 co-expression	Unfavorable	[225]
Hodgkin’s lymphoma	PD-L1 amplification	Unfavorable	[121]
DLBCL	↑ PD-L1 expression	Unfavorable	[216, 227]
NK/T-cell lymphoma	↑ PD-L1 expression	Unfavorable	[226]
Multiple myeloma	↑ Soluble PD-L1	Unfavorable	[223, 224]
All tumor types			
Meta-analyses	↑ PD-L1 expression	Unfavorable	[221, 228, 229]

*HER2* human epidermal growth factor receptor, *TNBC* triple-negative breast cancer, *NSCLC* non-small cell lung cancer, *SCLC* small cell lung cancer, *DLBCL* diffuse large B-cell lymphoma, *NK* natural killer cells

## Open questions for future research

Despite the progress in genetic and epigenetic regulation of PD-L1 expression, several gaps in the literature should be covered by intensive laboratory-based research. For instance, the signaling transduction pathways involved in PD-L1 regulation are only partially understood. Better understanding of the signaling mechanisms could provide the biologic rationale for combined targeted therapy with immunotherapy strategies in cancer. Furthermore, little is known about the post-translational modifications of PD-L1 protein including tyrosine or serine/threonine phosphorylation, acetylation, ubiquitination, and SUMOylation. It is also largely unknown how possible post-translational modifications not only regulate PD-L1 levels in the tumor cells, but also how they might affect its physiologic function or its interaction with the PD-1 receptor. In addition to PD-L1, the non-genetic mechanisms underlying PD-L2 expression and function in solid tumors and hematologic malignancies should be investigated, as both ligands compete for the same receptor, PD1, and therefore the relative levels of both proteins may impact certain immunotherapy approaches.

Regarding clinical practice, regulatory authorities both in Europe (European Medicine Agency), and the United States (Food and Drug Administration) have approved the use of PD-1/PD-L1 inhibitors for a variety of malignancies regardless of the presence or absence of predictive biomarkers. Exceptions include the use of pembrolizumab at the first and second line of NSCLC, which requires PD-L1 expression levels of ≥50% and ≥1% respectively, as well as the site agnostic indication for mismatch repair deficient tumors. In addition, the financial burden of the generalized use of these agents is considerable even in high-resource settings [230]. Overcoming this obstacle and achieving the personalized use of these agents requires a stepwise approach: first, taking into account the previously mentioned shortcomings of PD-L1 as a potential biomarker, it is important to retrospectively identify, in the large amount of collected tumor material from prospective studies, novel predictive biomarkers. These would ideally be prospectively validated, although the logistics of repeating single agent trials might be prohibitive. Instead, these biomarkers could form the basis of the next-generation combinatorial trials, of trials addressing the as yet unanswered question of the optimal duration of treatment or of trials in earlier disease settings where the overtreatment of already cured individuals in a massive scale could pose a significant public health burden.

## Summary

Despite the clinical success of immune checkpoint inhibition in many tumors through PD-L1/PD-1 blockade, relatively little is known regarding the biology of these regulators of cancer immune surveillance. Many mechanisms have been demonstrated to regulate the expression of PD-L1 including signaling pathways, transcriptional factors, and post-transcriptional modulators. The oncogenic signaling pathways such as JAK/STAT, RAS/ERK, or PI3K/AKT/MTOR are activated by gene mutations and growth factors. At the transcriptional level, a number of transcriptional factors seem to regulate PD-L1 expression including HIF-1, STAT3, NF-κΒ, and AP-1. PD-L1 is subject to post-transcriptional regulation by several miRNAs, CSN5, CMTM6, CDK4 and possibly other, still unknown mechanisms. Better understanding of PD-L1 regulation may pave the way for combinational treatments with both immune checkpoint inhibitors and targeted therapies against kinases or transcription factors many of which are already available for clinical use.
